# Smoking cessation behaviors and reasons for use of electronic cigarettes and heated tobacco products among Romanian adults

**DOI:** 10.1038/s41598-022-09456-7

**Published:** 2022-03-31

**Authors:** Sumaira Hussain, Chandrashekhar T. Sreeramareddy

**Affiliations:** Division of Community Medicine, International Medical University, Kuala Lumpur, Malaysia

**Keywords:** Disease prevention, Public health

## Abstract

We report cessation behaviors, reasons for use of electronic cigarettes (EC) and heated tobacco products (HTP) and association of their use with quit attempts and smoking intensity using Romania Global Adult Tobacco Survey 2018. Weighted estimates of EC and HTP by cigarette smoking (CS) status were assessed. Quit attempts, intention to quit, reasons for lack of intention to quit among current CS, and reasons for current use of EC and HTP were estimated. The association of ‘ever use’ of EC and HTP with cigarette smoking intensity and quit attempts was explored using binary logistic regression. Of the total 4571 surveyed, 1243 (27.3%) were current CS, 300 (24.4%) made quit attempts in the past 12 months. Only 38 (12.5%) and 26 (8.6%) had used EC and HTP as an aid to quit. Among current CS, 512 (41.2%) had no intention to quit. Reasons for this were, ‘enjoy smoking’ (86.1%), ‘reduce stress’ (65.9%), and ‘staying alert’ (46.3%). Awareness and use of EC and HTP were significantly higher among current CS. ‘Dual use’ of EC and HTP with CS was manifolds higher than stand-alone use. Reasons for current use of EC and HTP were ‘enjoyment’, and ‘use in places where smoking was prohibited’.

## Introduction

As early as 1940, the causal association between cigarette smoking (CS) and lung cancer was made^1^. CS is a risk factor for a host of diseases, including heart disease, diabetes, stroke, lung disease, and cancer^2^ attributing to about eight million deaths globally each year^3^. Furthermore, with the ongoing Covid-19 pandemic, there is evidence to support that smoking is associated with worse clinical outcomes of COVID-19 infection^4^. Globally the prevalence of CS has gradually declined^5^. Just when a target for ‘tobacco endgame’ was set by World Health Organization(WHO)^6^, novel tobacco products such as electronic cigarettes (EC) followed by heated tobacco products (HTP) emerged and they are being marketed as harm-reduction substitutes^7,8^. This coupled with their easy accessibility has brought about a significant change in the dynamics of tobacco use epidemiology since the regulatory policies for EC and HTP vary widely across the countries^9^.

Evidence is unclear if use of EC, also referred to as vaping, are useful in assisting cessation of CS^10^ but EC are often initiated to quit CS^11^. However, emerging evidence reports that EC use among current CS increases future smoking initiation among youth, increases the odds of transition to poly-tobacco use (PTU)^12,13^ among current CS and acts as a gateway to CS among non-smokers^14,15^. Recently, HTP use has also been reported to be increasing in some countries^17–19^. HTP are also promoted as a harm reduction product to aid in smoking cessation^8^. Although EC and HTP are marketed as less harmful substitutes to conventional cigarettes, they are still potentially harmful to human health, propelling the debate on their utility^7,9,20,21^.

The reported smoking cessation behaviors in European countries are far from desirable with less than a third of smokers having made a quit attempt (successful or not) in the previous 12 months^22^ and only about 40% of the current smokers had used nicotine replacement therapy or non-vaping products as an aid to quit smoking during their most recent quit attempt^23^. Quit rates are higher when using nicotine-containing EC compared to nicotine-replacement therapy^24^. However, this was not the primary reason for use of HTP and EC, which include, desire to reduce the number of cigarettes, suitable for use in areas of smoking prohibition and the perception that they are less harmful^25,26^. Examining cessation behaviors of cigarette smokers from nationally representative data will provide information to formulate regulatory policies on the novel tobacco products and provision of smoking cessation services, especially in Romania which lacks these.

Romania, a signatory to the WHO Framework Convention on Tobacco Control (FCTC)^27^, has launched a national campaign, “*the First Generation without Tobacco*”^28^, that endorses the WHO ‘tobacco end game’ by 2035^6^. Regardless, the prevalence of current CS remained high at 34.0% (females: 29.5% and males: 38.7%) in 2017^29^. As the use of EC and HTP is rising in European countries^30,31^, Romania follows the trend with ‘ever use’ of EC at 8.8% in 2014^30^ and ‘ever use’ of HTP at 4% in 2018^31^. Increasing use of these newer and apparently safer tobacco products makes it imperative to understand cigarette smoking cessation behaviors^32,33^ and reasons for use of EC and HTP^11,25^. The most recent GATS in Romania (2018) includes detailed questions about the use of EC and HTP. We aimed to study: (1) smoking cessation behaviors among current smokers, (2) the distribution of EC and HTP use behaviors according to cigarette smoking status, which includes current, former, and never cigarette smokers, and (3) the reasons for current use of EC and HTP. We also explored the association of ‘ever use’ of EC and HTP with cigarette smoking intensity and quit attempts.

## Methods

Romania Global Adult Tobacco Survey 2018 obtained ethical approvals from the Centre for Disease Control, Atlanta, United States, and the National Institute of Public Health. Informed consent for participation was obtained from each selected participant. If the participant was aged 15–17, consent was taken from the parent or the guardian. A separate ethical approval was not required since de-identified publicly available data was used to prepare this report. The study was carried out in compliance with the Declaration of Helsinki and followed all relevant guidelines and regulations.

### Data source

A secondary data analyses of Romania, Global Adult Tobacco Survey, 2018 was done. GATS is a nationally representative household survey of adults 15 years old and above that monitors adult tobacco use and tracks key tobacco use indicators. GATS uses a standardized methodology. It includes information on respondents’ background characteristics, tobacco use (cigarette smoking, smokeless tobacco, e-cigarettes, and heated tobacco products), cessation behaviors, exposure to secondhand smoke, economics, media, and knowledge, attitudes, and perceptions towards tobacco use. In Romania, GATS was conducted in 2018 by the National Institute of Public Health, and Totem Communication under the coordination of the Romanian Ministry of Health and WHO Country and Europe Regional Office. A multi-stage, geographically clustered sample design was used to produce nationally representative data. A total of 5,408 households were sampled. One individual was randomly chosen from each selected household to participate in the survey. The household response rate was 90.6%, the person-level response rate was 97.1%, and the overall response rate was 88.0%. There was a total of 4,571 completed individual interviews. Survey information was collected using handheld devices. Detailed methods of GATS are published elsewhere^34^.

### Outcome variables

The outcome variables were defined based on the response options to questionnaire items in Romania GATS 2018 and are summarised in Appendix A. The main outcome variables were ‘awareness’ about EC and HTP, ‘ever use’ of EC and HTP, ‘current’ CS, ‘current’ use of EC and HTP, ‘former’ CS, and ‘former’ use of EC and HTP. Briefly, ‘awareness’ was defined if the response was ‘yes’ to the questions “*have you ever heard of electronic cigarettes?*” *and* “*have you ever heard of heated tobacco products?*”*.* Questions regarding the use of EC and HTP were asked to only those adults who were ‘aware’ of EC and HTP. ‘Ever use’ was defined if response was ‘yes’ to questions “*have you ever, even once, used an electronic cigarette?*” *and* “*Have you ever, even once, used a heated tobacco product?*”*.* ‘Current use’ was specified if response was either ‘daily’ or ‘less than daily’ to questions, “*do you currently smoke tobacco?*”*,* “*Do you currently use electronic cigarettes?*’ *and* “*Do you currently use heated tobacco products?*”*.*

**Figure 1:**
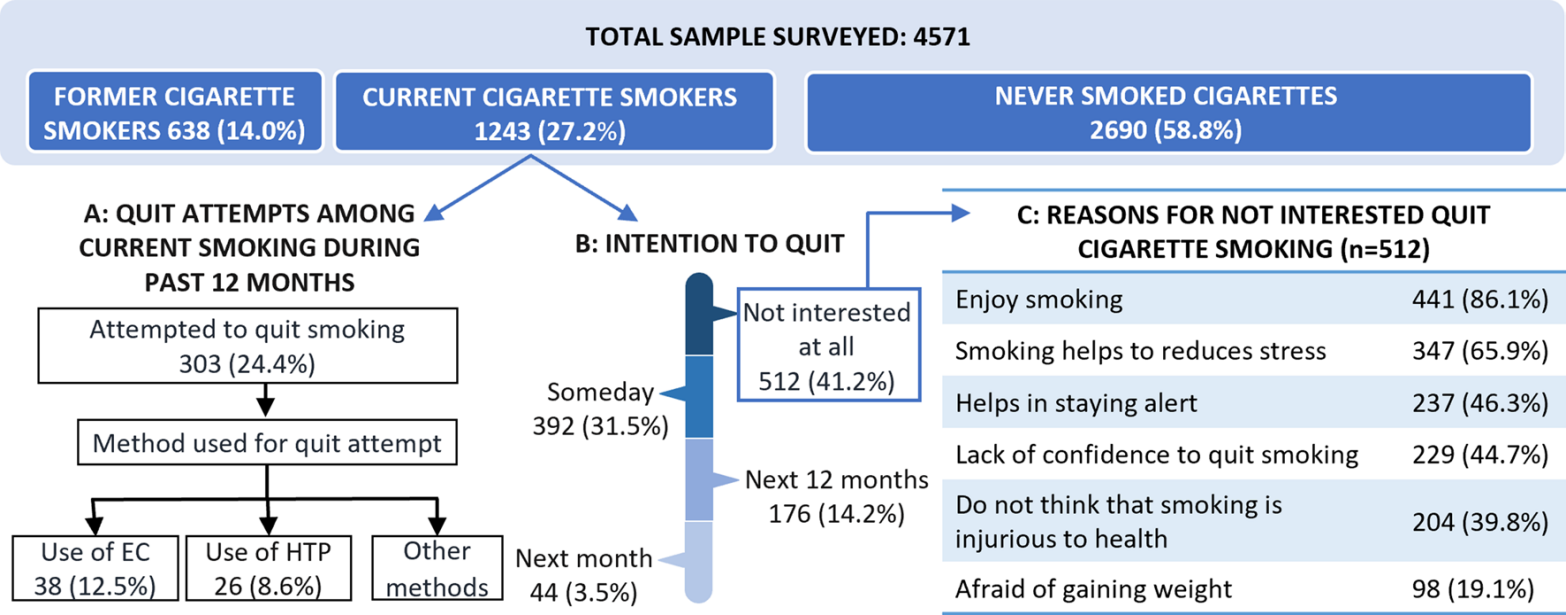
Intentions attempts to quit, and reasons for ‘no intention to quit among current CS’.

‘Former’ CS or ‘former’ EC use and HTP use was defined if response was ‘yes’ to questions, “*have you smoked tobacco daily in the past?*”*,* “*have you ever used electronic cigarettes daily in the past?*” *and* “*Have you ever used heated tobacco products daily in the past?*”*.* The intention to quit among ‘current’ CS was determined with, “*which of the following best describes your thinking about quitting smoking?*”. Those who responded, “*not interested in quitting*” were asked, “*which of the following are major reasons for why you are not interested in quitting smoking?*” (Fig. 1). Current EC and HTP users were asked “*which of the following are reasons that you use electronic cigarettes/ heated tobacco products?*”. The responses were recorded as ‘yes’, ‘no’ or ‘refused’ for identical options given to both EC and HTP.

### Predictor variables

The predictor variable definitions were based on the response options to the GATS questionnaire; they can be divided into sociodemographic factors and tobacco-associated factors. Operationalization predictor variables are shown in Appendix B. Sociodemographic factors are type of residence (urban/rural); sex; age group (15–24, 25–40, 41–55, 56–70 and ≥ 71); educational attainment [no education/primary, secondary, high school and higher education (college, university)]; wealth category [divided into quintiles from 1 (poorest) to 5 (richest)] based on the principal component analyses of a list of household assets; and primary occupation in the last 12 months [government employee, non-government or self-employed, student/homemaker and others (retired, unemployed, unable to work)]. The operationalization of tobacco-related variables is summarized in Appendix B. They include: number of sticks smoked per day (≤ 10, 10–20 and ≥ 20); rules about smoking at home as ‘permitted’ (allowed), ‘prohibited’ (allowed with exceptions and not allowed) and ‘no rules’; knowledge score about health complications of smoking (maximum possible score was 12); sources of exposure to information about dangers of CS, EC and HTP, (exposed to ‘at least one’ or ‘none’); and sources of exposure to promotional materials about CS and HTP (‘at least one’ or ‘none’).

### Statistical analyses

Descriptive statistics of raw and weighted numbers and proportions, means, and weighted prevalence estimates and their 95% confidence intervals (95% CI) were calculated. Survey weights were used to account for the complex sampling design of GATS. Socio-demographic and tobacco-related factors associated with current CS and ‘ever use’ of EC and HTP were determined by binary logistic regression using ‘*svy*’ command. Adjusted odds ratios (aOR) and their 95% CI were estimated. A p-value < 0.05 was considered statistically significant. All analyses were done on Stata/MP version 11.

### Ethics approval

Ethical boards of National Institute of Public Health, Romani and Centre for Disease Control, Atlanta, USA.

## Results

The socio-demographic characteristics of the respondents are shown in Table 1 as raw numbers, weighted numbers and percentages. Among the adults surveyed, the highest proportions were observed from female respondents (51.6%), aged 25–55 years (55%), urban residence (56.9%), educated up to high school or higher (66.9%), occupational category of non-governmental or self-employed (39.3%), and wealth category of poorest and poorer (46.2%).

**Table 1:** Raw numbers, weighted numbers and proportions of socio-demographic characteristics of adults in Romania GATS 2018.

	Raw number (%)	Weighted number (%)
**Type of residence**		
Urban	2318 (50.7)	10,496,537 (56.9)
Rural	2253 (49.3)	7,955,787 (43.1)
**Sex**		
Male	2111 (46.2)	8,939,290 (48.5)
Female	2460 (53.8)	9,513,034 (51.6)
**Age groups**		
15–24	343 (7.5)	2,171,286 (11.8)
25–40	997 (21.8)	5,439,022 (29.5)
41–55	1066 (23.3)	4,707,914 (25.5)
56–70	1376 (30.1)	4,220,688 (22.9)
≥ 71	789 (17.3)	1,913,414 (10.4)
**Educational attainment**		
No/primary	428 (9.5)	1,197,463 (6.6)
Secondary	1346 (29.9)	4,807,753 (26.5)
High school	1812 (40.3)	8,032,612 (44.2)
Higher education	916 (20.4)	4,126,121 (22.7)
**Wealth category**		
Poorest	985 (21.6)	4,718,235 (25.6)
poorer	731 (16.0)	3,799,781 (20.6)
Middle	600 (13.1)	2,742,219 (14.9)
richer	1160 (25.4)	3,877,398 (21.0)
Richest	1095 (24.0)	3,314,692 (18.0)
**Occupation**		
Government employee	423 (9.3)	2,090,359 (11.4)
Non-governmental or self-employed	1461 (32.2)	7,200,990 (39.3)
Student/homemaker	741 (16.3)	3,593,117 (19.6)
Others (retired, unemployed, unable to work)	1916 (42.2)	5,438,952 (29.7)

### Tobacco use behaviors

Weighted prevalence (%) estimates of tobacco use behaviours among all the adults surveyed are shown in Table 2. The total current use of CS is at 30.2% (95% CI: 28.1, 32.3), with EC use higher than HTP at 3.4% (95% CI: 2.6, 4.1). Awareness for EC at 76.4% (95% CI:74.3, 78.6) was more than double for HTP, which corresponds to more of the population having tried using EC at 7.8% (95% CI: 6.8, 8.9) when compared to HTP. The mean age at initiation of CS was generally lower at 21.1 years (95% CI: 16.8, 25.4), compared to EC and HTP, who attracts a somewhat older audience. The mean knowledge scores about the health complications of smoking were similar across individuals aware of CS, EC, and HTP.

**Table 2:** Tobacco product use behaviors and knowledge about health effects of CS among adults in Romania GATS 2018.

Prevalence	EC (95% CI)	HTP (95% CI)	CS (95% CI)
Awareness (Wt. prev. & 95% CI)	76.4 (74.3, 78.6)	30.1 (27.6, 32.6)	–
Current use (Wt. prev. & 95% CI)	3.4 (2.6, 4.1)	1.3 (0.9, 1.7)	30.2 (28.1, 32.3)
Former use (Wt. prev. & 95% CI)	3.2 (2.5, 3.8)	0.6 (0.3, 0.9)	12.5 (11.2, 13.8)
Ever use (Wt. prev. & 95% CI)^£^	7.8 (6.8, 8.9)	3.0 (2.2, 3.8)	42.7 (44.8, 40.5)
Age at initiation (Mean, 95% CI)	32.6 (26.4, 38.8)^¥^	36.3 (29.5, 43.2)^¥^	21.1 (16.8, 25.4)^×^
Knowledge score (Mean, 95% CI)^€^	8.91 (8.69, 9.14)	8.47 (8.12, 8.82)	8.09 (7.77, 8.42)

^£^Ever CS was those who has ever smoked during lifetime i.e., current, and former CS.

^×^Age at which initiated daily smoking.

^¥^Age at which tried EC or HTP even once.

^€^Estimated for only current CS and aware of EC and HTP.

### Comparison of EC and HTP by cigarette smoking status

The distribution of EC and HTP awareness and use by CS status are shown in Table 3. The use of EC and HTP (even once) was much higher among individuals who were currently CS compared to those not involved in CS (former CS, never CS). For example, the proportion who were current EC users was 8.13, 1.25, and 0.37 among current CS, former CS, and never CS respectively. Most notable was the current ‘dual use’ of tobacco products, which includes use of CS along with either EC or HTP, which is 8.1% and 3.6% respectively. Polytobacco use (CS + EC + HTP) was 2.9%.

**Table 3:** Proportions (%) of EC and HTP awareness and use by adults according to their CS status (current, former, and never).

	Entire sample	Current CS (n = 1243)	Former CS (n = 638)	Never CS (n = 2690)	p-value
**EC**					
Awareness	70.8	87.4	80.1	61.0	< 0.001
Ever use	6.8	18.8	6.9	1.2	< 0.001
Current users	2.6	8.1	1.3	0.4	< 0.001
**HTP**					
awareness	23.7	37.9	24.0	17.1	
Ever use	2.3	6.0	2.2	0.7	< 0.001
Current use	1.1	3.6	0.6	0.04	< 0.001
Quit ratio (former CS/ever CS)	[638/ (1243 + 638)] = 0.34				

### Smoking cessation behaviors, and reasons for “no intention to quit” CS

An overall quit ratio for CS (Former CS/ Ever CS) was 0.34 (Table 3). Figure 1 shows the distribution of the survey sample by cigarette smoking status, smoking cessation behaviors, and reasons for ‘no intention to quit’. Of the total 4571 surveyed, 1243 (27.3%) were involved in current CS. Only 300 (24.4%) of current CS had ‘*tried to quit smoking*’ during the past 12 months. Of these 300, only 38 (12.5%) and 26 (8.6%) had tried using EC and HTP respectively to quit CS (Fig. 1A). Among current CS, 512 (41.2%) were ‘not interested at all’ in quitting smoking (Fig. 1B). The common reasons cited for this were ‘*like to smoke*’ (86.4%), ‘*smoking helps to reduce stress*’ (65.9%), ‘*smoking helps to keep alert*’ (45.1%), ‘*do not have the confidence that they can quit*’ (44.7%) and ‘*do not believe smoking is injurious to health*’ (39.8%) (Fig. 1C). Weighted numbers and percentages are shown in e-Table 1.

### Raw numbers, proportions, and reasons for current use of EC and HTP

**Figure 2:**
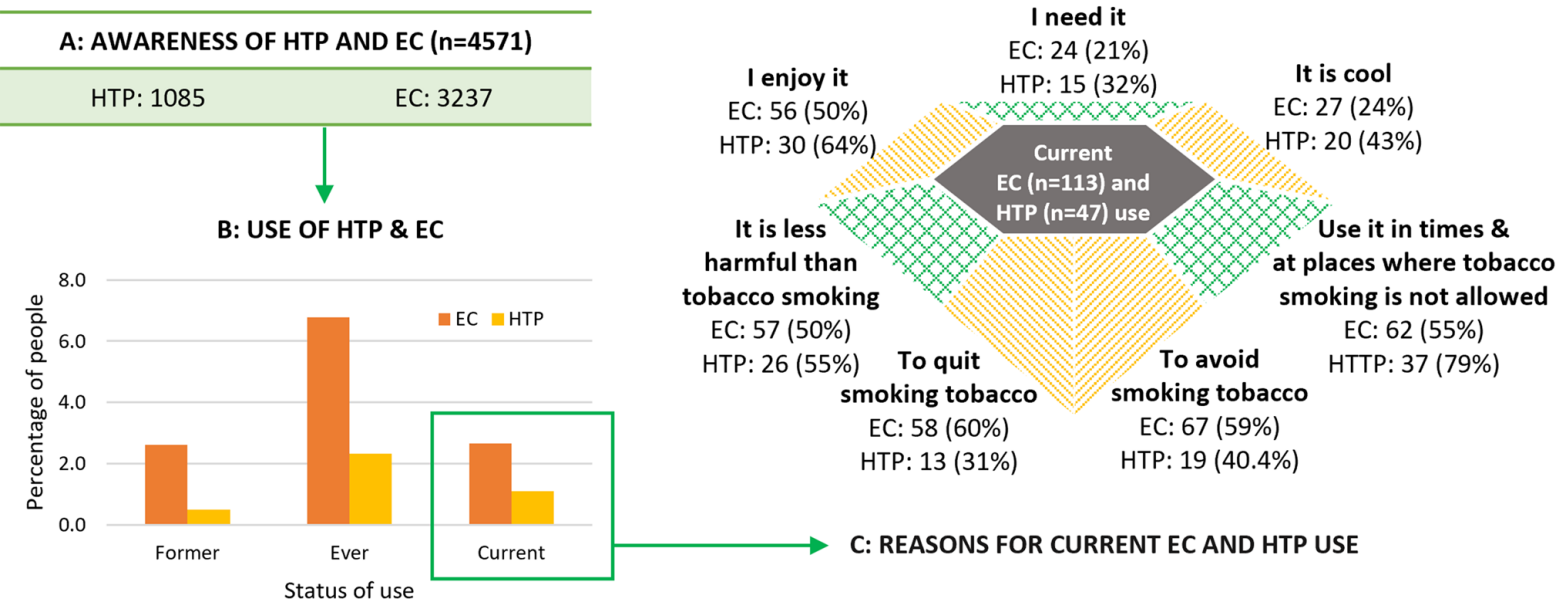
Awareness, current use, and reasons for current use of EC and HTP.

Figure 2 provides raw numbers about awareness of EC and HTP, their usage, and reasons for use among ‘current’ users. Of the 4571 adults surveyed, 3271 (70.8%) and 1085 (23.7%) were aware of EC and HTP respectively (Fig. 2A). Of those aware 113 (3.7%) and 47 (2.0%) were current users of EC and HTP respectively (Fig. 2B). Key reasons cited for current EC use were ‘*can be used when or in places when smoking cigarettes was prohibited*’ (62.4%), ‘*to quit smoking*’ (58.9%), and ‘*to avoid smoking*’ (58.7%). The top reasons for current HTP use were: ‘*can be used when or in places when smoking was prohibited*’ (79.0%), ‘*enjoy using HTP*’ (67.9%), and ‘*less harmful than smoking*’ (58.7%). Weighted numbers and percentages are shown in e-Table 2.

### Factors associated with current CS and ‘ever use’ of EC and HTP

Table 4 shows the results of binary logistic regression for factors associated with current CS and ‘ever use’ of EC and HTP. After adjusting for individual and tobacco-related factors, current CS was associated with male sex, rules about smoking at home, exposure to information about dangers of smoking, cigarette promotional materials, and knowledge score about health complications of smoking (Table 4). Men had twice higher odds (aOR: 2.1, 95% CI: 1.7, 2.7) of engaging in current CS than women. Adults from homes where smoking was prohibited had 0.2 times lower odds (95% CI: 0.1, 0.2) of CS than those from homes where smoking was permitted. Adults exposed to at least one source of information about the dangers of smoking and higher knowledge score of health complications of smoking had 0.8 (95% CI: 0.6, 1.0) and 0.9 (95% CI: 0.9, 1.0) lower odds of engaging in CS whereas those individuals exposed to at least one cigarette promotional material had 1.7 higher odds (95% CI: 1.3, 2.1) of participating in CS compared to adults who were not exposed to any.

**Table 4:** Factors associated with current CS, ever use EC and HTP.

	Current Cigarette Smoking		E-cigarette Ever Use		Ever Use of HTP	
	Adj. OR (95%CI)	p-value	Adj. OR (95%CI)	p-value	Adj. OR (95%CI)	p-value
**Residence**						
Rural	1		1		1	
Urban	1.1 (0.8, 1.4)	0.516	1.7 (1.1, 2.7)	0.027	2.3 (1.0, 5.4)	0.063
**Sex**						
Female	1		1		1	
Male	2.1 (1.7, 2.7)	< 0.001	1.3 (0.8, 2.1)	0.308	1.2 (0.5, 3.1)	0.623
**Age groups**						
15–24	1		1		1	
25–40	1.0 (0.7, 1.5)	0.949	0.8 (0.4, 1.5)	0.424	0.5 (0.2, 1.3)	0.137
41–55	1.1 (0.8, 1.7)	0.56	0.6 (0.3, 1.3)	0.179	1.0 (0.3, 3.1)	0.985
56–70	0.7 (0.5, 1.2)	0.221	0.4 (0.2, 1.1)	0.074	0.3 (0.0, 2.0)	0.218
≥ 71	0.1 (0.1, 0.3)	< 0.001	0.6 (0.1, 4.3)	0.638		
**Educational attainment**						
No/primary	1		1		1	
Secondary	1.6 (0.9, 3.0)	0.123	7.1 (0.8, 67.7)	0.087	-	
High school	1.1 (0.6, 2.0)	0.853	11.1 (1.1, 109.6)	0.039	3.7 (1.1, 11.6)	0.028
Higher education	1.1 (0.5, 2.2)	0.817	13.5 (1.3, 142.7)	0.03	3.2 (0.9, 11.7)	0.077
**Wealth category**						
Poorest	1		1		1	
Poorer	1.1 (0.8, 1.5)	0.425	1.9 (1.0, 3.5)	0.038	0.7 (0.3, 1.5)	0.334
Middle	1.5 (1.0, 2.0)	0.025	2.1 (1.1, 4.0)	0.029	0.5 (0.1, 2.1)	0.344
Richer	1.3 (0.9, 1.8)	0.176	1.4 (0.7, 2.6)	0.34	0.0 (0.0, 0.3)	0.001
Richest	1.4 (0.9, 2.1)	0.097	2.7 (1.2, 6.1)	0.013	0.3 (0.0, 2.1)	0.207
**Occupation**						
Government employee	1		1		1	
Non-govt/self-employed	1.2 (0.9, 1.7)	0.245	1.2 (0.6, 2.5)	0.585	1.4 (0.5, 4.7)	0.531
Student/homemaker	0.6 (0.4, 0.9)	0.017	1.4 (0.6, 3.6)	0.452	4.0 (1.0, 15.2)	0.042
Others ×	0.6 (0.4, 1.0)	0.056	0.5 (0.2, 1.3)	0.168	4.4 (0.9, 22.1)	0.07
**Rules about smoking at home**						
Permitted	1		1		1	
Prohibited	0.2 (0.1, 0.2)	< 0.001	0.8 (0.5, 1.2)	0.232	0.7 (0.3, 1.4)	0.293
No rules	0.3 (0.2, 0.4)	< 0.001	0.7 (0.3, 1.6)	0.391	1.5 (0.3, 8.5)	0.613
**Knowledge score about health complications of smoking**						
	0.9 (0.9, 1.0)	< 0.001	1.0 (0.9, 1.3)	0.26	0.9 (0.8, 1.0)	0.15
**Exposure to information about dangers of CS**						
None	1		1		1	
At least one source	0.8 (0.6, 1.0)	0.02	0.9 (0.6, 1.5)	0.815	0.8 (0.3, 2.0)	0.675
**Exposure to information about promotional materials about cigarettes**						
None	1		1		1	
At least one source	1.7 (1.3, 2.1)	< 0.001	1.2 (0.8, 1.9)	0.36	1.5 (0.7, 3.3)	0.277
**Quit smoking attempts made during last 12 months**						
No			1		1	
Yes			1.8 (1.0, 3.0)	0.033	1.4 (0.6, 3.6)	0.436
**Number of sticks (cigarettes or other smoking) smoked per day**						
< 10			1		1	
11–20			1.2 (0.7, 1.9)	0.472	0.7 (0.3, 1.6)	0.372
> 20			2.5 (1.2, 5.1)	0.015	1.2 (0.3, 4.6)	0.76
**Exposure to information about dangers of EC**						
At least one source			1			
None			1.0 (0.6, 1.7)	0.904		
**Exposure to information about dangers of HTP**						
At least one source					1	
None					1.3 (0.4, 4.2)	0.638
**Exposure to information about promotional materials about HTP**						
At least one source					1	
None					0.9 (0.4, 2.1)	0.82

× retired, unemployed, unable to work etc.

After adjusting for individual and tobacco related factors, ‘ever use’ (even once) of EC or HTP was associated with area of residence, wealth, educational attainment, attempt to quit smoking, and number of cigarettes smoked per day (Table 4). Individuals from urban areas had 1.7 (95% CI: 1.1, 2.7) higher odds of ever use of EC than those from rural areas. Similarly, the richest individuals had 2.7 (95% CI: 1.2, 6.1) higher odds for ‘ever use’ of EC and richer individuals had 0.01 times lower odds for ever use of HTP (aOR 0.01, 95% CI: 0.01, 0.3) compared to the poorest. Individuals who had attained higher education had 13 times higher odds for ‘ever use’ of EC (aOR 13.5, 95% CI: 1.3, 142.7) whereas adults educated up to high school education had 3.7 times higher odds for ‘ever use’ of HTP (aOR 3.7, 95% CI: 1.1, 11.6). Adults who had tried to quit cigarette smoking in the past twelve months had 1.8 (95% CI: 1.0, 3.0) higher odds of ‘ever use’ of EC. Individuals who smoked more than 20 cigarettes a day had 2.5 (95% CI: 1.2, 5.1) higher odds of ‘ever use’ of EC.

## Discussion

Secondary data analysis of the GATS 2018 survey revealed that nearly a third of Romanian adults currently smoke cigarettes, which has not declined from GATS 2011. Only a quarter of current CS had made a quit attempt during the past 12 months. More than a third had ‘no intention to quit, mainly because they ‘like to smoke’, ‘smoking reduces stress’ and ‘smoking helps keep alert’. Use of EC and HTP was several folds higher among individuals practicing current CS than never CS or former CS. Current ‘dual use’ of either EC and CS or HTP and CS was common, rather than the use of EC or HTP alone. Use of either EC or HTP was seldom used as a smoking cessation aid by those who tried to quit. The perception that EC and HTP are ‘less harmful than CS’ and ‘can be used in places where CS was prohibited’ were common reasons for the current use of EC and HTP. Ever use of EC was associated with the attempt to quit CS and smoking > 20 cigarettes per day.

GATS 2018 shows that CS has increased relative to previous GATS 2011 and comparable survey reports^29,35^. However, notably, mean sticks smoked per day (16.3, 95% CI 15.5, 17.0) remained unchanged from GATS 2011. GATS 2018 provides more robust estimates of EC and HTP than European surveys^16,30^. Though awareness and ever use of EC have increased, the current use of EC is still low. With HTP being a more recent product, estimates of awareness, ever, and current use were lower and are comparable to previous surveys^16^. Regardless of the marketing of EC and HTP as harm reduction agents and aides to quit CS, the quit ratio (former CS/ ever CS) was only 0.3 in GATS 2018 and the proportions of current CS that had made ‘quit attempts’ was low. EC and HTP use were used as an aid to quit CS among < 10% of current CS during their ‘quit attempt’. Comparable rates of EC and HTP as an ‘aid to quit smoking’ were reported from other countries^32,36^. Recommended methods to quit smoking such as counseling, nicotine replacement therapy, quitline, and prescription medicines were also utilized by < 10% and 2/3rd of them had tried to ‘*quit without assistance*’^35^. A similar pattern of cessation methods used by current smokers was reported from European countries^36^. Attempts to quit smoking without any assistance call for prioritizing provision recommended cessation methods services to reduce the current pool of CS also as a tobacco control measure. The general lack of intention to quit among current CS can be explained by the health belief model^37^. The reasons for ‘*no intention to quit*’ such as ‘*lack of confidence to quit*’ (self-efficacy), ‘do not believe that CS is injurious to health’ (perceived severity), and poorer knowledge on harms among current CS underscores that need for health promotion interventions to improve health behaviors. Romanian tobacco regulations are well within a broad framework of WHO-FCTC^38^, however, quitting services and their uptake appears suboptimal^35^.

Our results showed that EC and HTP were used to overcome smoke-free policies that are strictly implemented in Romania. A high proportion of ‘dual users’ and reasons cited for use of EC and HTP suggest that EC or HTP are used by current CS to fulfill ‘nicotine cravings’ in smoke-free situations. CS and EC are prohibited in public transport^39^. However, the use of EC at work and public places is still allowed and current regulations on HTP do not exist^38^. Varying levels of exposure to secondhand aerosols of EC in European countries were reported in indoor areas explains the EC and HTP use in places where CS is prohibited^39^. EC and HTP are also used for reasons other than quitting CS^25^. In Romania, only half considered EC and HTP as harmful. More than 50% of smokers perceived e-cigarettes to be equally same or more harmful than conventional cigarettes^40^. Regardless of smokers’ perception of harmfulness, extended use of any type of tobacco product should be discouraged. Replacement by safer alternatives and reduction in smoking intensity does not discount the risk of early death^9^.

After adjusting for socio-demographic factors, ‘quit attempt’ and smoking > 20 sticks per day had higher odds of being ever user of EC. This suggests possible prior unsuccessful attempts to quit smoking with aid of EC perhaps from user perception that ECs are less harmful. ‘Smoke-free’ rules at home, knowledge about health complications, and exposure to information about dangers of smoking had lower odds of CS, implying that greater awareness and strict rules about CS at home had lower odds of CS. Smoke-free home rules have been shown to reduce the smoking intensity in 20 GATS countries^41^. In GATS 2018, Romanian adults were aware of about eight of the 10 health effects of smoking, and nearly two-thirds were exposed at least to one source of information about the dangers of smoking^35^. GATS-based report has shown that anti-smoking messages on the media increase the knowledge about the harms of smoking^42^. However, exposure to promotional materials about cigarettes increased the odds of CS. Despite the general ban on the advertisements with an exception for point-of-sale of tobacco products^38^, a third of the adults reported having seen promotional materials in stores selling cigarettes (not limited to tobacco stores exclusively) and on the internet^35^.

### Policy implications

Romania GATS 2018 results provide a policy platform for improving upon the provision of existing tobacco cessation methods and emphasizing dissemination of anti-smoking messages for all types of tobacco products to promote and increase the demand for cessation behaviors among current users of cigarettes as well as EC and HTP. In Romania, EC and HTP were primarily used for reasons other than as aids to smoking cessation, like other European countries^25,43^. Current tobacco regulations must be made more comprehensive to include stricter regulations on EC and to introduce regulation on HTP^44^. Furthermore, EC and HTP must be included in smoke-free policies as per European Tobacco Products Directive (TPD)^44^. Stringent implementation of regulation regarding exposure to pro-tobacco sales at the point of sales and via the internet is required^45^. As there is growing evidence to support that smoking is associated with worse clinical outcomes of COVID-19 infection, it brings to the forefront the need to evaluate effective methods for tobacco cessation, including EC and HTP^4,46^. However, the containment measures imposed during the pandemic have had varying responses on tobacco use behaviors, resulting in conflicting reports of increased smoking and smoking cessation. Regardless, there needs to be further information disseminated about smokers as a high-risk group for Covid-19^46^.

### Strengths and limitations

The strengths of our report are a high response rate (88%) and a nationally representative large sample that provides more accurate estimates for Romania than other regional surveys^16,29,47^. However, self-reported tobacco use behaviors are known to have social desirability bias leading to underestimation^48^. As the GATS survey is a cross-sectional design, changes in tobacco use behaviors could not be reported, such as long-term abstinence, quit rates and switching between tobacco products. The association of ‘ever use’ of EC and HTP lacked statistical power due to smaller sample sizes. Deeper insights into reasons for the lack of intention to quit CS and the current use of EC and HTP could not be provided due to limited options in the GATS questionnaire. Qualitative exploration is required to understand user perceptions about tobacco products^49^.

## Conclusion

Romania GATS 2018 showed that cigarette smoking cessation behaviors were poor and CS prevalence was consistently high as in previous years. The entry of EC and HTP into the market has led to the emergence of ‘dual use’ and ‘poly tobacco use’ among current CS. EC and HTP were not mainly used to quit smoking but to circumvent smoke-free policies. Comprehensive tobacco control policies inclusive of EC and HTP are required. Measures to increase the demand for and provision of supportive smoking cessation services should be implemented to achieve a ‘tobacco-free generation.’

## Supplementary Information

Supplementary Information.
