# Supplementary material for: Smoking cessation behaviors and reasons for use of electronic cigarettes and heated tobacco products among Romanian adults
Source: Sci Rep. 2022 Mar 31;12:5446. doi: 10.1038/s41598-022-09456-7 (PMC8968304; doi:10.1038/s41598-022-09456-7)
Supplement: Supplementary file 1 — Supplementary Information. [file 41598_2022_9456_MOESM1_ESM.docx]

Appendix A

**Operationalization of outcome variables**

| **Question** | **Response options** |
| --- | --- |
| 1. Do you currently smoke tobacco? | Daily basis, less than daily and not at all |
| 1. How old were you when you first started smoking tobacco daily? | Open response |
| 1. In the past, have you smoked tobacco? | Yes/No |
| 1. Have you smoked tobacco daily in the past? | Yes/No |
| 1. Which of the following best describes your thinking about quitting smoking? | - Quit within the next month - Thinking within the next 12 months - Quit someday, but not next 12 months - not interested in quitting |
| 1. During the past 12 months, did you use any of the following to try to stop smoking tobacco? Counseling including at a smoking cessation clinic?  - Counseling, including at a smoking cessation clinic? - Nicotine replacement therapy, such as the patch or gum - Other prescription medications, for example Zyban, Champix? - Natural plant products? - A quit line or a smoking telephone support line? - Acupuncture? - Electronic cigarettes? - Heated tobacco products like iQOS or GLO? - Quit without assistance? | Yes/No |
| 1. Prior to today, have you ever heard of electronic cigarettes? | Yes/No |
| 1. Have you ever, even once, used an electronic cigarette? | Yes/No |
| 1. Do you currently use electronic cigarettes? | Daily basis, less than daily and not at all |
| 1. Have you ever used electronic cigarettes daily in the past? | Yes/No |
| 1. I want to ask you about products that heat, but do not burn tobacco like iQOS and GLO. Prior to today, have you ever heard of heated tobacco products? | Yes/No |
| 1. Have you ever, even once, used a heated tobacco product? | Yes/No |
| 1. Do you currently use heated tobacco products? | Daily basis, less than daily and not at all |
| 1. Have you ever used heated tobacco products daily in the past? | Yes/No |

Appendix B

**Operationalization of tobacco-related factors (predictor variables)**

| **Question** | **Response options** |
| --- | --- |
| 1. On average, how many of the following products do you currently smoke each day: manufactured cigarettes, hand-rolled cigarettes, pipes full of tobacco, cigars/cigarillos, waterpipe sessions, any others? | Absolute numbers |
| 1. Which of the following best describes the rules about smoking tobacco inside of your home? | Allowed, not allowed but exceptions, never allowed and no rules. |
| 1. Based on what you know or believe, does smoking cause the following: stroke, myocardial infarction, lung cancer, emphysema, stomach cancer, mouth cancer, periodontal disease, bone loss, premature birth, erectile dysfunction, tuberculosis, diabetes? | Yes, No, Don’t know |
| 1. In the last 30days, have you noticed information about the dangers of smoking cigarettes or that encourages quitting in any of the following places: newspapers/magazines, television, radio, billboards, internet and somewhere else? | Yes, No and Not Applicable |
| 1. In the last 30days, have you noticed information about the dangers of using electronic cigarettes in any of the following places: newspapers/magazines, television, radio, billboards, internet and somewhere else? | Yes, No and Not Applicable |
| 1. In the last 30days, have you noticed information about the dangers of using heated tobacco products like iQOS or GLO in any of the following places: newspapers/magazines, television, radio, billboards, internet and somewhere else? | Yes, No and Not Applicable |
| 1. In the last 30days, have you noticed any advertisements or signs promoting cigarettes in the following places: stores, internet and anywhere else? | Yes, No and Not Applicable |
| 1. In the last 30days, have you noticed any advertisements or signs promoting heated tobacco products like iQOS or GLO in the following places: stores, internet and anywhere else? | Yes, No and Not Applicable |

**Operational definition predictor variables**

|  | **Response item** |
| --- | --- |
| 1. Tobacco products smoked per day | Sum of tobacco products in item 1 is operationalized as ≤10, 11-20 and >20 tobacco products per day. |
| 1. Rules about smoking at home | In item 2, Allowed is operationalised as ‘permitted’, Allowed with exceptions, and not allowed is operationalised as ‘prohibited’ and No rules remains No rules. |
| 1. Knowledge score about health complications of smoking | Scoring was given to each health complication in Item 3 (Yes = 1, No = 0, Don’t know = 0). Total sum was knowledge score. |
| 1. Exposure to information about dangers of smoking | ‘’Yes” to each source in Item 4 was added. This was operationalised as: at least one and none. |
| 1. Exposure to information about dangers of e-cigarettes | ‘’Yes” to each source in Item 5 was added. This was operationalised as: at least one and none. |
| 1. Exposure to information about dangers of heated tobacco products | ‘’Yes” to each source in Item 6 was added. This was operationalised as: at least one and none. |
| 1. Exposure to information about promotions about cigarettes | ‘’Yes” to each source in Item 7 was added. This was operationalised as: at least one and none. |
| 1. Exposure to information about promotions about heated tobacco products | ‘’Yes” to each source in Item 8 was added. This was operationalised as: at least one and none. |

**e-table 1 Reasons for ‘current use’ of EC and HTP**

| Reasons for using | **E-cigarettes (n=113)** | | **HTP (n=47)** | |
| --- | --- | --- | --- | --- |
|  | Raw numbers (%) | Weighted numbers (%) | Raw numbers (%) | Weighted numbers (%) |
| To quit smoking tobacco | 58 (60.4) | 302,840 (58.9) | 13 (31.0) | 53,829 (28.7) |
| To avoid smoking tobacco | 67 (59.3) | 345,744 (58.7) | 19 (40.4) | \|88,070 (40.7) |
| I can use it at times when or in places where tobacco smoking is not allowed. | 62 (54.9) | 367,478 (62.4) | 37 (78.7) | 170,911 (79.0) |
| It is less harmful than smoking tobacco | 57 (50.4) | 292,876 (49.7) | 26 55.3) | 127,049 (58.7) |
| Because it is cool (fashionable) | 27 (23.9) | 161,557 (27.4) | 20 (42.6) | 91,599 (42.4) |
| Because I enjoy it. | 56 (49.6) | 307,057 (52.1) | 30 (63.8) | 146,765 (67.9) |
| Because I need it (addicted) | 24 (21.2) | 150,723 (25.6) | 15 (31.9) | 82,655 (38.2) |

**e-table 2 Reasons for ‘no intention to quit’ smoking**

| **Reason for not quitting Cigarette smoking** | Raw numbers (%) | Weighted numbers (%) |
| --- | --- | --- |
| You are addicted to smoking and do not think you can quit. | 229 (44.7) | 1,012,781 (41.82) |
| You do not think smoking is bad for your health. | 204 (39.8) | 1,001,749.3 (41.4) |
| Smoking helps to reduce your stress. | 347 (67.8) | 1,597,015.9 (65.9) |
| Smoking keeps you alert. | 237 (46.3) | 1,091,387 (45.1) |
| You like to smoke. | 441 (86.1) | 2,092,737 (86.4) |
| You are afraid of gaining weight if you quit. | 98 (19.1) | 455,794.46 (18.8) |
